# Cumulative effects of climate and landscape change drive spatial distribution of Rocky Mountain wolverine (*Gulo gulo* L.)

**DOI:** 10.1002/ece3.3337

**Published:** 2017-09-21

**Authors:** Nicole Heim, Jason T. Fisher, Anthony Clevenger, John Paczkowski, John Volpe

**Affiliations:** ^1^ University of Victoria Victoria BC Canada; ^2^ InnoTech Alberta University of Victoria Victoria BC Canada; ^3^ Western Transportation Institute Montana State University Bozeman MT USA; ^4^ Alberta Environment and Parks Edmonton AB Canada

**Keywords:** human footprint, interspecific interactions, mesocarnivore, occupancy, species distribution

## Abstract

Contemporary landscapes are subject to a multitude of human‐derived stressors. Effects of such stressors are increasingly realized by population declines and large‐scale extirpation of taxa worldwide. Most notably, cumulative effects of climate and landscape change can limit species’ local adaptation and dispersal capabilities, thereby reducing realized niche space and range extent. Resolving the cumulative effects of multiple stressors on species persistence is a pressing challenge in ecology, especially for declining species. For example, wolverines (*Gulo gulo* L.) persist on only 40% of their historic North American range. While climate change has been shown to be a mechanism of range retractions, anthropogenic landscape disturbance has been recently implicated. We hypothesized these two interact to effect declines. We surveyed wolverine occurrence using camera trapping and genetic tagging at 104 sites at the wolverine range edge, spanning a 15,000 km^2^ gradient of climate, topographic, anthropogenic, and biotic variables. We used occupancy and generalized linear models to disentangle the factors explaining wolverine distribution. Persistent spring snow pack—expected to decrease with climate change—was a significant predictor, but so was anthropogenic landscape change. Canid mesocarnivores, which we hypothesize are competitors supported by anthropogenic landscape change, had comparatively weaker effect. Wolverine population declines and range shifts likely result from climate change and landscape change operating in tandem. We contend that similar results are likely for many species and that research that simultaneously examines climate change, landscape change, and the biotic landscape is warranted. Ecology research and species conservation plans that address these interactions are more likely to meet their objectives.

## 1 INTRODUCTION

Species range limits are a primary focus of ecological and evolutionary research (Gaston, 2009; Sexton, McIntyre, Angert, & Rice, 2009). As spatial manifestations of a species’ niche and dispersal abilities (Hargreaves, Samis, & Eckert, 2013), they are dictated by multiple niche axes and hence limited by multiple interacting environmental conditions. These conditions are projected to change markedly under the twin drivers of landscape change and climate change. Contemporary landscapes are affected by a myriad of human‐derived stressors (Halpern, Selkoe, Micheli, & Kappel, 2007; Venter et al., 2006), which may interact additively, synergistically, or antagonistically (Crain, Kroeker, & Halpern, 2008), thus shifting species’ range limits and ultimately changing biodiversity (Chen, Hill, Ohlemuller, Roy, & Thomas, 2011; Maxwell, Fuller, Brooks, & Watson, 2016). Climate change is predicted to negatively impact global biodiversity (Walther et al., 2002), but also interacts with anthropogenic footprint, complicating the challenge of teasing cause from effect in understanding species declines and range shifts (Hansen et al., 2001; Oliver & Morecroft, 2014). Where habitat becomes fragmented, dispersal is reduced by biotic factors not incorporated into climate model predictions (Kubisch et al. 2013). Consequently, interspecific interactions significantly affect species range limits (Louthan, Doak, & Angert, 2015), and these interactions are themselves both subject to, and agents of, biodiversity losses (or gains) from landscape and climate change (Pecl et al., 2017). Both scientific understanding and effective conservation management require disentangling of the relative effects of landscape and climate change on (e.g., Sultaire et al., 2016).

Resolving mechanisms of decline from among cumulative effects is one of the most pressing challenges in applied ecology and conservation (Sala et al., 2000). Despite global attention to adverse effects of human footprint on biodiversity (Sanderson et al., 2002), actual species conservation and recovery are slow. For example, almost half of Canadian “special concern” species have deteriorated since 1977 (Favaro et al., 2014). Recovery failures can be attributed to weak political will (Waples, Nammack, Cochrane, & Hutchings, 2013), but another mechanism is scientific in nature: debates over mechanisms of decline (Brook, Sodhi, & Bradshaw, 2008; Hutchings, Butchart, Collen, Schwartz, & Waples, 2012). There is growing recognition that multiple stressors acting additively or synergistically cause species declines (Brook et al., 2008; Côté, Darling, & Brown, 2016; Darling & Côté, 2008), but few studies disentangle the relative importance of multiple natural, climatic, and anthropogenic factors; most of these focus on bird communities (Howard, Stephens, Pearce‐Higgins, Gregory, & Willis, 2015; Oliver & Morecroft, 2014). A single‐mechanism focus naturally results from traditional experimentation paradigms (Hurlbert, 1984) but leaves a lingering question: How do landscape, climate, and biotic communities interact to effect distribution patterns and population declines? We contend that these processes are most likely to manifest at a species’ range margin. Therefore, we use a spatially expansive distribution dataset—one that encompasses a species’ dynamic range margin—to test for the influence of climate and landscape stressors, using wolverines (*Gulo gulo luscus*, 1758) as a model.

Wolverines have wide global distribution but populations are continuing to decline (IUCN 2016). In North America, wolverines have been extirpated from over 40% of their historical range (Laliberte & Ripple, 2004). Wolverines once ranged across Canada from west to east into the northern regions of United States. However, their current range south of the boreal forest has retracted westward to the east slopes of the Canadian Rocky Mountains. A recent withdrawal of the petition to list wolverines under the US Endangered Species Act was based on controversy around a single‐stressor climate‐change model, exemplifying the multistressor problem (The Wolverine Foundation 2014). This proposal failed in part because the mechanisms of decline remain complex, equivocal, with principle arguments rooted in climate‐based threats deemed not imminent (DEFENDERS OF WILDLIFE vs. SALLY JEWELL et al 2016).

Past studies (e.g., Aubry, McKelvey, & Copeland, 2007; Krebs et al. 2007) suggest wolverines select rugged, high‐elevation landscapes with persistent spring snow cover hypothesized to provide for stable maternal dens and cold food storage in warmer regions (Copeland et al., 2010; Inman, Magoun, Persson, & Mattisson, 2012). Consequently, range retraction and declines have been attributed to declining winter snowpack due to climate change (Brodie & Post, 2010), although with debate (DeVink, Berezanski, & Imrie, 2011). Recently, evidence from boreal forests suggests that wolverine distribution is not restricted by spring snow cover (Webb et al., 2016).

Additionally, wolverines have long been subject to harvest pressure (Squires, Copeland, Ulizio, Schwartz, & Ruggiero, 2007) and avoid human‐caused landscape change, such as road density and resource extraction (Rowland et al., 2003; Krebs et al. 2007; Fisher et al., 2013). Anthropogenic disturbance resulting in landscape change can reduce species’ survivorship or range via resource loss; alter interspecific interactions; reduce gene flow and genetic complexity; and increase susceptibility to stochastic events (Dunning, Danielson, & Pulliam, 1992; Fahrig, 2003; Landa, Strand, Swenson, & Skogland, 1997). Areas of limited land use may provide refugia from anthropogenic disturbance (Krebs & Lewis, 1999; Stewart et al. 2016). As a scavenging carnivore, wolverines may also select habitat to reduce negative interspecific interactions with intraguild carnivores less adapted to navigate alpine terrain (Inman et al., 2012; Mattisson, Andrén, Persson, & Segerström, 2011). Discerning the relative contribution of these factors for wolverines—as with many species—has been difficult, as it requires examination of a population spanning multiple potential stressors, habitat types, and climatic conditions.

To address this challenge, we sampled wolverines in the Canadian Rocky Mountains, a region comprising heterogeneous habitat, topography, and snowpack. The east slopes of the Rocky Mountains are subject to rapid forest loss (Global Forest Watch Canada 2014) and house a diverse carnivore community. We hypothesized that, peripheral populations on the edge of this species’ longitudinal spatial range, both climate and landscape changes interact to limit distribution, and thus are implicated in range contractions. We also hypothesized that competing species may additionally be implicated in wolverine declines. We tested these hypotheses by surveying wolverine occurrence across a large gradient of climatic variability and landscape disturbance. We modeled wolverine occurrence against biophysical variables describing (1) natural landcover, (2) human‐caused disturbance factors, (3) climatic and abiotic factors, (4) co‐occurrence of intraguild carnivores, and (5) the cumulative effects of these biophysical and anthropogenic factors. We predicted that the cumulative effects of climate, landscape, and competitors simultaneously limit wolverine distribution at their range edge.

## 2 METHODS

### 2.1 Study area

The Canadian central Rocky Mountains are comprised of alpine, subalpine, and montane natural subregions (Natural Regions Committee 2006, Figure 1). The alpine above treeline is dominated by low‐growing vegetation adapted to harsh climatic conditions. The subalpine is dominated by Engelmann spruce (*Picea englemannii*) and subalpine fur (*Abies lasiocarpa*). Lower elevation montane is dominated by Douglas fir (*Pseudotsuga menziesii*), trembling aspen (*Populus tremuloides*), and lodgepole pine (*Pinus contorta*). Topography is rugged; elevations range from 1,400 m to over 2,400 m.

**Figure 1:**
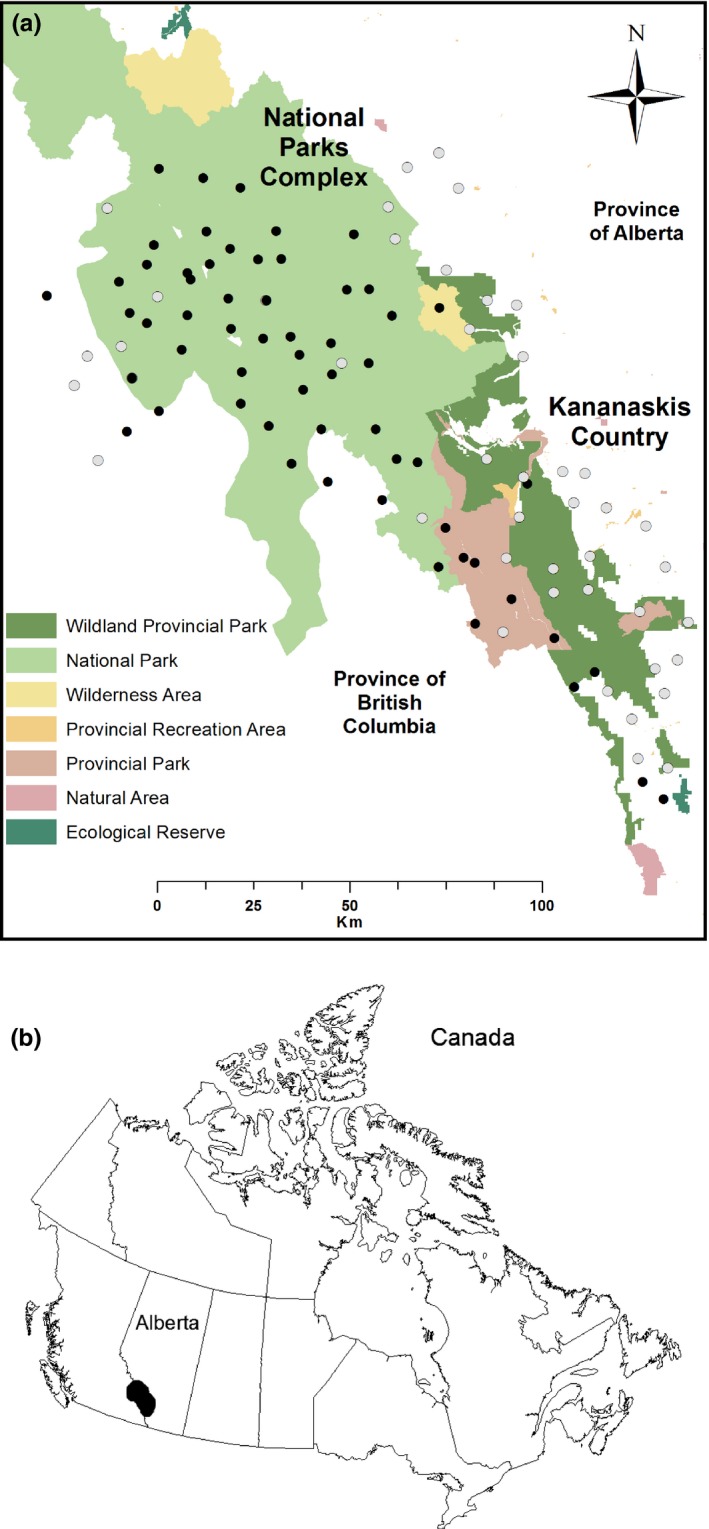
(a) Study area map showing wolverine sample sites (black points = wolverine detected, light gray points = wolverine not detected) located throughout the south‐central Canadian Rocky Mountain range, extending west‐east from British Columbia, through a gradient of protected areas and land management designations that include a National Parks Complex and Alberta Parks Kananaskis Country region, and out along the eastern slopes of Alberta.

This study spans a complex of landscapes with varying degrees of legal land‐use designations. All experience human use to some degree, but the intensity and spatial distribution of use varies across the whole area. Within the National Parks Complex (NPC)—Banff, Yoho, and Kootenay National Parks, which span alpine to low montane subregions—there are highways and a railway, nonmotorized recreational trails, three ski resorts, and two tourist towns with millions of visitors per year. These impacts vary across the NPC, with intense disturbance clustered in some places and none in others. Within the Kananaskis Country region (KC)—an equally mountainous region grading into foothills—there is a mosaic of varying degrees of landscape protection; some places are legislated by the same level of protection as NPC or higher, whereas others permit various combinations of oil and gas exploration, mining, timber harvest, livestock grazing, motorized and nonmotorized recreation, urban development, and fur trapping. The entire study area is within wolverines’ putative range as described by historical and contemporary fur trapping records (Webb, Manzer, Anderson, & Jokinen, 2013) and local observations of wolverine sign.

### 2.2 Sampling design

We used a systematic study design (Fisher et al., 2013) that divided the 15,000 km^2^ landscape into 104, 12 × 12 km^2^ grid cells (43 in KC; 61 in NPC; Figure 1), reflecting the minimum home‐range size of female wolverines (Banci, 1994). Within each cell, we selected one sampling site to maximize detection probability; statistical inference occurs at the scale of the grid‐cell, and therefore, site placement is not expected to affect inference (MacKenzie, 2006). Sites were a minimum of 6,000 m apart. This same design has been used in multiple‐related studies (Fisher, Anholt, & Volpe, 2011; Fisher et al., 2013; Fisher and Bradbury 2014, Stewart et al., 2016).

### 2.3 Species sampling

**Figure 2:**
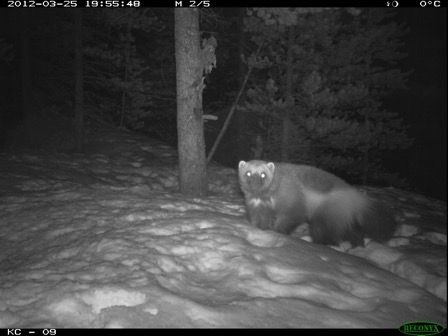
Photograph of a wolverine detected using a remote camera trap located in the Kananaskis Country region of the central Canadian Rocky Mountains

We sampled wolverine and other carnivore occurrence in winter 2010–2013 using noninvasive genetic tagging (NGT) and camera trapping (Figure 2). NGT (Waits & Paetkau, 2005) allows individual identification, but can be subject to detection bias wherein animals present at a site do not deposit hair (Dreher, Rosa, Lukacs, Scribner, & Winterstein, 2009; Fisher & Bradbury, 2014). Combining camera trapping (Burton et al., 2015; O'Connell, Nichols, & Karanth, 2011) with NGT trapping provides an additional sampling technique and permits estimation of NGT detection bias (Fisher & Bradbury, 2014; Fisher, Heim, Code, & Paczkowski, 2016).

At each site, a single Reconyx RM30 or PM30 infrared‐triggered digital camera faced the hair trap—a tree wrapped loosely with barbed wire, baited with a frozen beaver carcass and scent lure (O'Gorman's, Montana, USA). Cameras were programmed at high sensitivity, five images per trigger, 1 s apart, and rapid fire with no delay. We collected photo and hair samples monthly January–April. Our study area was a polygon around the outermost cameras in the array, and the sampling site is the area around the trap potentially imaged by the camera (Burton et al., 2015). The data frame consisted of *n* = 104 sites, surveyed *t* = 3 times.

### 2.4 Genetic analysis of wolverine individuals

The USFS Rocky Mountain Research Station, Missoula, Montana, USA, extracted DNA from hair using QIAGEN DNeasy Blood and Tissue kits with modifications for hair sampling (Mills, Pilgrim, Schwartz, & McKelvey, 2000) and assayed with a 16 locus mtDNA microsatellite panel (Schwartz et al., 2009). Samples were examined at a 344 bp region of the left domain of the mtDNA control region using primers and protocols detailed in Schwartz et al. (2007), a region of the genome proven variable in other wolverine genetic studies (Wilson et al. 2000, Walker et al. 2001, Schwartz 2007). DNA sequence data were obtained using the Big Dye kit and the 3700 DNA Analyzer (ABI; High Throughput Genomics Unit, Seattle, WA, USA). DNA sequence data were viewed and aligned with Sequencher (Gene Codes Corp. MI) and compared for wolverine haplotype using program Dambe (Xia 2013).

DNA from hair samples was initially tested using three microsatellite loci that amplify well in wolverine from noninvasive samples: Gg4 and Gg7, (Davis and Strobeck 1998) and Ggu101 (Duffy et al. 1998). Hair samples that amplified using these three loci were analyzed using 13 additional microsatellite loci: Gg3, Ma2, Ma8, Ma9, Tt1, and Tt4 (Davis and Strobeck 1998); Ggu216, Ggu234, Ggu238 (Duffy et al. 1998), Mvis020 (Flemming et al. 1999), Mvis72, Mvis075 (Flemming et al. 1999), and Lut604 (Dallas and Piertney 1998). The samples were also tested using an SRX/SRY analysis to determine sex (Hedmark et al., 2004). Data were error checked using program Dropout (McKelvey and Schwartz 2005).

### 2.5 Camera image analysis for species distribution models

We downloaded camera images monthly and identified each image to species. We calculated a conservative measure to describe temporal persistence at a site we termed “wolverine frequency”: the repeated occurrence of generic wolverines (not individually identified) among three, one‐month survey periods, and yielding a 0–3 count variable. Sampling across the greater study area was achieved over consecutive winter seasons (2010–2013), with wolverine frequency for each site calculated for a single season. This measure serves to minimize effects of temporal vagaries in detection rates (Burton et al., 2015). As camera data are subject to less detection bias than NGT data (Fisher & Bradbury, 2014), we used camera data only to model wolverine‐habitat selection against abiotic and biotic variables.

### 2.6 Quantifying landscape variables for species distribution models

A species’ relationship to its habitat is scale dependent (Wiens 1989, Wiens et al. 1993), and the best‐fit scale can be empirically estimated (Elith & Leathwick, 2009; Holland, Bert, & Fahrig, 2004). Multiscale analyses of wolverine‐habitat selection identified scales from 700 m radius to 5,000 m and 7,600 m (Krebs et al. 2007, Fisher et al., 2011). Following Fisher et al. (2011) we imposed 20 circular buffers (500‐m to 10‐km radius) around each sampling site in ArcGIS 9.3.1 (ESRI, Inc.). Within each buffer, we quantified the percent landcover (*i.e*., landscape composition) from a 16‐class landcover dataset based on LandSat imagery and a digital elevation model (DEM; McDermid et al., 2009). We calculated topographic ruggedness index (TRI; Riley, DeGloria, & Elliot, 1999) from a DEM. We quantified the percent area covered by persistent spring snow (measured as years out of 12 with spring snow between April 14 and May 15) using Moderate Resolution Imaging Spectroradiometer (MODIS) satellite data, following Copeland et al. (2010). We measured anthropogenic features (ABMI Human Footprint Map 2010) in 12 composite classes and grouped into two main categories: % area of block features (urban or industrial disturbance); and density (km/km^2^) of linear features (roads, cutlines, pipelines, seismic lines, motorized, and nonmotorized recreational trails). Landscape variables retained for analyses are described in Table 1.

**Table 1:** List and description of variables hypothesized to explain the spatial pattern of wolverine occurrence across the south‐central region of the Canadian Rocky Mountains

Model set	Variable name	Variable code	Description	Hypothesized direction of effect
Landcover	Dense conifer	DENSECON	>70% crown closure; >80% coniferous	+
	Mixed forest	MIXED	21%–79% coniferous	−
	Open conifer	OPENCON	<30% crown closure; >80% coniferous	−
	Shrub	SHRUB	Shrub cover	+
	Herb	HERB	Herb cover	Neutral
	Regeneration	REGEN	Regenerating portions of the landscape (cutblocks, burns etc.)	−
	Snow and Ice	SNOW.ICE	Perennial, or permanent, snow and ice cover	+
Human Footprint	Urban block‐shaped features	BLOCKURB	Urban setting (towns, developed recreational lease areas)	−
	Linear roadways	LINROAD	Paved and unpaved transportation features (local roads, highway, and railway)	−
	Industrial linear features	LININD	Linear industrial cutlines (pipeline, transmission, and seismic lines)	−
	“Quiet” recreational linear trails	LINRECQ	Quiet linear recreational features (i.e., designated hiking trails)	Neutral
	“Loud” recreational linear trails	LINRECL	Loud linear recreational features (designated ATV and snowmobile trails)	−
Climatic‐abiotic	Topographic Ruggedness Index	TRI	Topographic ruggedness index, average elevation difference in a given area	+
	Persistent spring snow	SP.SNOW	Number of years (out of 12) an area was snow covered during spring months	+
Biotic	Wolf occurrence frequency	WOLF	Number of wolf detections	−
	Cougar occurrence frequency	COUG	Number of cougar detections	−
	Coyote occurrence frequency	COYO	Number of coyote detections	Neutral
	Lynx occurrence frequency	LYNX	Number of lynx detections	Neutral
	Bobcat occurrence frequency	BOBC	Number of bobcat detections	Neutral
	Fox occurrence frequency	FOX	Number of red fox detections	Neutral
	Marten occurrence frequency	MART	Number of American marten detections	Neutral

### 2.7 Data exploration

We standardized (X – μ/σ) independent variables to compare effect sizes, used Pearson correlation coefficient (*r* ^2^) matrices and variance inflation factor (VIF) estimation to identify and assess the extent of collinearity (Zuur et al. 2010; Zuur, Hilbe, & Ieno, 2013). We retained variables with VIF < 5. A cut‐off value of VIF < 3 is preferred (Craney and Surles 2002); however, a cut‐off value of VIF < 5 enabled inclusion of predicted and ecologically meaningful variables for the full global model while minimizing *r* ^2^ values <.50. Some remaining correlation exists (see Pearson correlation matrix of landcover class variables in Appendix S2); however, our modeling approach was expected to correct for erroneous results of shared variance. Two collinear variables were retained varying in their relative biological importance and habitat association: SNOW.ICE, SP.SNOW, Table 1), Landcover class indexes perennial snow and ice (McDermid et al., 2009), whereas persistent spring snow is an annual average measure of ephemeral snow cover (Aubry et al., 2007; Inman et al., 2012; Magoun & Copeland, 1998; Schwartz et al., 2009).

### 2.8 Species distribution models

Modeling serial detection data is an area of active research without current consensus (Banks‐Leite et al., 2014; Burton et al., 2015; Rota, Fletcher, Dorazio, & Betts, 2009). For example, the 0s in a 101 detection history can be considered detection error—imposed by an animal being temporarily unavailable for detection (Efford & Dawson, 2012), or an ecological signal, as an index of frequency of site use, rendering occupancy models inappropriate. We employed a dual approach to analysis (Banks‐Leite et al., 2014; Burton et al., 2015) and looked for convergence in results.

First, we mapped longitudinal changes in wolverine distribution, corresponding to the gradient in landscape disturbance, using occupancy models. These treat 0s in serial detection histories as potential false absences, a noted problem in species surveys (MacKenzie et al., 2002), including camera‐trap surveys (Burton et al., 2015). Hierarchical occupancy models estimate the probability of detection (*p*) of a species—if present—and the probability of species occupancy at a site, given *p*. Occupancy models are analogous to as simultaneous generalized linear models (GLMs) of serial detection data, applied to each component of the model, with binomial errors (logistic link). We created custom single‐season occupancy models in Presence v.4.4 software (Hines, 2006), with estimated occupancy and detection probability held constant. Closed occupancy models assume (1) species occupancy changes are random, and (2) extant species have a nonzero probability of detection within the survey; our design fit these assumptions.

Second, we assumed that 0s are part of the ecological signal contained in the “wolverine frequency” metric (0–3 months of site use) and so tested hypotheses about wolverines’ response to landscape features using generalized linear models (GLMs; poisson errors, log link; Zuur et al., 2013) in software package R version 3.0.2 (R Core Team 2014). We investigated violation of model assumptions using diagnostic plots (Matthiopoulos 2011; Zuur et al., 2013). Of these diagnostics, we assessed models for overdispersion (which may arise from capped counts and/or model misspecification) but found none. To identify the best spatial scale for analysis, we followed Fisher et al. (2011). We created a global model of variables measured at each of the 20 spatial scales and used the stepAIC function in R package MASS (Venables & Ripley, 2002) to identify the best‐supported model based on Akaike information criterion (AIC) scores, and AIC weights—normalized AIC scores between 0 and 1, analogous to the probability a model in the candidate set is the best‐supported model (Burnham & Anderson, 2002). The 10‐km scale was best supported (see Heim, 2015 for more information), so we used climate and landscape variables quantified at that scale for all subsequent analyses.

We adopted a nested approach to model selection that allowed us to test our five main hypotheses across multiple different models. Each hypothesis was represented by a candidate model set (Table 2, Results): natural landcover, human disturbance, climatic‐abiotic, biotic features, and cumulative effects. Each hypothesis could be represented by a number of different models within a set; for example, the “natural landcover” hypothesis might be driven mostly by mixedwood forest (model 4), or shrub land (model 6), or combinations of features (model 1). We ranked AIC scores for each model and calculated AIC weights for models *within* each candidate set. The best‐supported model from each candidate set—representing one of each of the five main hypotheses—was then competed against one another based on AIC scores. AIC weights were calculated for this set of best models to determine relative support for each hypothesis.

**Table 2:** Wolverine‐habitat model selections in the south‐central region of the Canadian Rocky Mountains. The best‐fit model for each model set is indicated by ΔAIC = 0.00 and AIC weight closest to 1.00. AIC values are calculated within each model set

Model set	Model no.	Variables	Residual deviance	Residual *df*	AIC	ΔAIC	AIC weight	−2LL
Null	0		149.46	90	286.23			
Landcover	**1**	**DENSECON + MIXED + + OPENCON + SHRUB + HERB + REGEN + SNOW.ICE**	**65.01**	**83**	**213.78**	**0.00**	**1.00**	**199.78**
	2	DENSECON + OPENCON	125.70	88	264.48	50.7	0.00	260.48
	3	DENSECON	147.73	89	284.51	70.73	0.00	282.51
	4	MIXED	126.12	89	262.90	49.12	0.00	260.90
	5	SHRUB + HERB	144.46	88	283.23	69.45	0.00	279.23
	6	SHRUB	151.90	89	288.67	74.89	0.00	286.67
	7	REGEN	134.76	89	271.53	57.75	0.00	269.53
	8	SNOW.ICE	105.03	89	241.80	28.02	0.00	239.80
Human Disturbance	9	BLOCKURB + LINROAD + LININD + LINRECQ + LINRECL	79.30	85	224.08	2.12	0.23	214.08
	10	BLOCKURB	150.92	89	287.69	65.73	0.00	285.69
	11	LINROAD + LININD + LINRECQ + LINRECL	82.86	86	225.63	3.67	0.11	217.63
	12	LINROAD	145.53	89	282.31	60.35	0.00	280.31
	**13**	**LININD**	**85.18**	**89**	**221.96**	**0.00**	**0.66**	**219.96**
	14	LINRECQ + LINRECL	116.38	88	255.16	33.2	0.00	251.16
Climatic‐Abiotic	15	TRI + SP.SNOW	93.71	88	232.49	1.32	0.34	228.49
	16	TRI	137.10	89	273.88	42.71	0.00	271.88
	17	SP.SNOW	94.39	89	231.17	0.00	0.66	229.17
Biotic	18	WOLF + COUG + COYO + LYNX + BOBC + FOX + MART	104.56	83	253.33	7.02	0.03	239.33
	19	WOLF + COUG	141.98	88	280.76	34.45	0.00	276.76
	20	WOLF + COUG + COYO	110.21	87	250.98	4.67	0.08	244.98
	21	LYNX + BOBC + COYO + FOX + MART	138.42	86	281.20	34.89	0.00	271.20
	**22**	**FOX + COYOTE**	**107.53**	**88**	**246.31**	**0.00**	**0.89**	**242.31**
	23	LYNX	150.58	89	287.35	41.04	0.00	285.35
Cumulative effects	**24**	**DENSECON + MIXED + SHRUB + HERB + REGEN + SNOW.ICE + BLOCKURB + LININD + LINRECL + SP.SNOW + COYOTE + FOX**	48.631	78	207.74	0.00	1.00	**183.74**

Models highlighted in bold represent the best‐fit out of each model set.

Cumulative model was included as its own set.

## 3 RESULTS

### 3.1 Wolverine individual identification and occupancy across the study area

The number of individual wolverines was significantly higher in the NPC compared to adjacent KC. A total of 53 wolverines were genetically identified and three additional individuals were identified from IRC data (as per Magoun et al. 2011), for a total of 56 individual wolverines. A total of 49 wolverines were detected within the NPC (33 males, 16 females), and only seven were in KC (two males, two females, and three unknown). Of >2,000 hair samples collected, 833 were attempted and 339 were genotyped for wolverine. The genotyping error rate was 0.26%, with a probability of identity (pID) = 1.30E^−10^ and probability of identity given siblings (pIDsib) = 4.01E^−05^.

**Figure 3:**
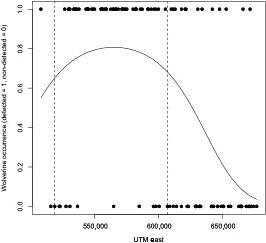
Estimated wolverine occupancy modeled with UTM east coordinates. Vertical dashed lines correspond to the western and eastern‐most boundaries of the National Parks Complex, refer to Figure 1(a) of study area and park boundaries (Map Datum: Nad 83, Zone 11)

There was a marked longitudinal gradient in wolverine occupancy (Figure 3). Estimated wolverine occupancy (ψ) in the NPC (ψ = 0.88, *SE* = 0.05, *p* = .4) was over double that estimated in the adjacent KC region (ψ = 0.36, *SE* = 0.11, *p* = .24). Although the highest occupancy occurred within the nationally protected areas, some sites within the NPC and >50% of the sites within the KC region did not detect wolverine (Figure 1, see Appendix S1).

### 3.2 Species distribution models

Among landcover‐only models, six landcover variables best‐explained wolverine frequency (AIC_w_ = 1.00, Table 2). Wolverines selected dense conifer, shrub, herb, snow and ice cover, and avoided regenerating and mixed forests. Among climatic‐abiotic models, wolverines selected persistent spring snow cover, which was better supported than topographic ruggedness (AIC_w_ = 0.66, AIC_w_ = 0.34; respectively, Table 2). Among human disturbance models, wolverines avoided linear industrial features. Among biotic models, wolverines were most strongly and negatively influenced by the occurrence of two meso‐canid carnivore species: coyote and fox (Tables 2 and 4).

However, the cumulative effects model better‐explained wolverine frequency than any single‐factor model (AIC_w_ = 0.95, Table 3). Within this model, landcover variables carried the greatest weight of evidence (ER = 19), followed by linear industrial features (ER = 6.33), with the strongest negative effect. Persistent spring snow was less supported (ER = 0.86); meso‐canid occurrence was least supported (ER = 0.35). Although the relative likelihood and strength of variables within the cumulative effects model describing wolverine frequency vary and some effects are quite weak (Table 4), 95% of weight of evidence supports the cumulative effects model (Table 3), suggesting no single factor alone is driving wolverine frequency.

**Table 3:** Best‐fit wolverine‐habitat models across each model set. Comparing across the best‐fit, or minimum adequate, models (1, 13, 17, 22) suggests that a combination of the natural and anthropogenic variables included in the cumulative effects model (24) best‐explain patterns of wolverine frequency

Model no., set	Variables	Res. deviance	Res. *df*	AIC	ΔAIC	AIC weight	−2LL
1, Landcover	DENSECON + MIXED + OPENCON + SHRUB + HERB + REGEN + SNOW.ICE	65.01	83	213.78	6.04	0.05	201.78
13, Human Disturbance	LININD	85.18	89	221.96	14.22	0.00	219.96
17, Abiotic	SP.SNOW	94.392	89	231.17	23.43	0.00	229.17
22, Biotic	FOX + COYOTE	107.53	88	246.31	38.57	0.00	242.31
**24, Cumulative Effects**	**DENSECON + MIXED + SHRUB + HERB + REGEN + SNOW.ICE + BLOCKURB + LININD + LINRECL + SP.SNOW + COYOTE + FOX**	**48.631**	**78**	**207.74**	**0**	**0.95**	**183.74**

Models highlighted in bold represent the best‐fit out of each model set.

**Table 4:** Lists estimated β‐parameters and supporting evidence for the variables included cumulative effects wolverine distribution model. Evidence ratios (ER) describe the relative likelihood of support for inclusion of one variable (or a group of variables) compared to the exclusion of the variable(s) in a global model (Burham and Anderson 1998). Example: The ER for the set of landcover variables shows 19 times more support in explaining wolverine‐habitat selection relative to other set(s) of variables

Parameter	Estimate	*SE*	*z*‐value	Pr (*z*)	ER
Intercept	−1.218	0.381	−3.201	0.001	
DENSE	0.605	0.200	3.023	0.002	
MIXED	−0.929	0.450	−2.062	0.039	
SHRUB	0.338	0.120	2.811	0.005	
HERB	0.363	0.230	1.579	0.114	
REGEN	−0.003	0.507	−0.005	0.996	
SNOW.ICE	0.048	0.026	1.842	0.065	19.00
BLOCKURB	0.002	0.112	0.022	0.983	
LINRECL	1.056	0.653	1.617	0.106	
LININD	−1.243	0.648	−1.919	0.055	6.33
SP.SNOW	0.409	0.320	1.279	0.201	0.86
RED FOX	−0.170	0.162	−1.051	0.293	
COYOTE	−0.306	0.297	−1.030	0.303	0.35

## 4 DISCUSSION

### 4.1 Wolverines were rare in areas of greater disturbance

There was a sharp demarcation at the current wolverine range margin, associated with increasing landscape development, diminishing snowpack, and a shift in mesocarnivore relative abundance. With only seven of the 49 individuals detected outside of the NPC, the marked decline in occupancy just outside the nationally protected areas was unexpected given that wolverine populations have historically and recently supported trapping throughout the study area (Poole & Mowat, 2001; Webb et al., 2013). KC is rugged, with large areas of persistent spring snow, and abundant ungulate and small mammal prey—key landscape characteristics for wolverine (Copeland et al., 2007; Krebs et al. 2007). The decreasing pattern of wolverine occupancy is not subtle (Figure 3) and represents a sharp spatial range boundary not far from the NPC border. Fisher et al. (2013) found a similar pattern to the north, wherein probability of wolverine occupancy and frequency plummeted in increasingly human disturbed landscapes despite the presence of apparently suitable habitat.

Although the wolverine decline manifests around this political boundary, the ecological mechanisms for this pattern—natural landcover changes, spring snowpack, and anthropogenic disturbance—transcend this one political boundary, varying instead across the entire study area. Evidence strongly suggests the spatial patterns of wolverine distribution and decline result from a cumulative response to both climate and landscape change, as quantified by variables measured in our analysis.

### 4.2 Wolverine distribution was best explained by cumulative effects

In the central Canadian Rockies, wolverine distribution was best explained by a combination of natural landcover, linear industrial features, persistent spring snow cover, and mesocarnivore (coyote and red fox) occurrence. Contrasting conclusions drawn from southern peripheries of their range where predominantly climate‐change mechanisms—diminishing spring snow—are implicated in wolverine declines, central Rockies populations are also affected by anthropogenic disturbance and associated changes to mesopredator communities. These spatial associations are strongly supported by evidence collected over a very large area comprising one of the largest North American wolverine studies extant and allow us to infer potential mechanisms.

Natural landcover variables provided the strongest effect on wolverine distribution. As expected, wolverine selected dense conifer cover, shrub and herb, and perennial snow and ice cover, reflecting habitat with abundant prey such as marmot (*Marmota*), Bighorn sheep (*Ovis canadensis*), and Mountain goat (*Oreamnos americanus*; Krebs et al. 2007; Lewis, Flynn, Beier, Gregovich, & Barten, 2012), and spatial refugia from competition (Copeland et al., 2007; Inman et al., 2012). We expect wolverines asymmetrically trade‐off between risk‐related foraging opportunities at lower elevations exposed to increased competition and predation from co‐occurring carnivores (Inman et al., 2012). Although behaviorally‐mediated space use and habitat selection influences prey distribution, (Lima & Dill, 1990; Sih, 2005), these same principles are rarely applied to predators (Durant, 1998).

Selection at higher elevation habitats by wolverine is also hypothesized to be driven by cold temperatures and deep snow packs within mountainous regions of the northern United States (Aubry et al., 2007; Copeland et al., 2010; Inman et al., 2012). Supporting these hypotheses, morphological characteristics—such as large feet—make wolverines adapted for efficient travel in northern snow‐covered biomes (e.g., Copeland & Whitman, 2003). In our study area, persistent spring snow cover was indeed a factor explaining wolverine distribution, as predicted (Aubry et al., 2007; Copeland et al., 2010). However, spring snow weakly explained wolverine‐habitat selection relative to linear industrial features—the most spatially extensive anthropogenic disturbance feature on this landscape. Likewise, wolverine distribution in the boreal forest a few 100 km's north of our study area is not constrained by persistent spring snow cover (Webb et al., 2016).

Although linear disturbances—highlighted as a key factor in our results—do not appear to impede wolverine movement, they are known to avoid extensive and intensive levels of anthropogenic disturbance such as major highway and road networks (May, Landa, van Dijk, Linnell, & Andersen, 2006; Krebs et al. 2007) and seismic lines (Fisher et al., 2013). The mechanism remains elusive, but we can make some inferences. Extensive linear infrastructure and associated disturbance contribute to the combined effects of landscape change (Primack, 2010). They increase access into areas by humans and competitively dominant wolves (Ciuti et al., 2012; Dickie, Serrouya, McNay, & Boutin, 2016; Latham, Latham, Boyce, & Boutin, 2011), and generalist coyotes and foxes which adapt and proliferate in altered environments (Laliberte & Ripple, 2004). Differential niche space requirements can promote spatial coexistence among intraguild carnivores where intraspecific competition is greater than interspecific competition (Murrell, Purves, & Law, 2002). However, spatial coexistence may be limited when prey preference is shared in a landscape that supports increased relative density of one species over another. In this case, spatially‐mediated competition will favor the more abundant competitor, irrespective of body size (Amarasekare, 2003). We hypothesize that the negative association between wolverines, coyote, and fox may be a result of expanding coyote and fox populations driven by anthropogenic landscape change along Alberta's eastern slopes and may be one mechanism driving the negative association with landscape change and anthropogenic (especially linear) landscape features. The effect of mesocarnivores we noted is relatively weak while the effect of landscape change is quite strong; but if mesocarnivore occurrence was correlated with human land‐use activities (beyond our detection limit) or if there is a time lag in the process of displacement of wolverines by more abundant competitors, then we would expect a weak signal. Given the importance of interspecific interactions for wolverines in Scandinavia (Mattisson et al., 2011; van Dijk et al., 2008), additional attention to these interactions is warranted in North America, as a mechanism additive to climate and landscape change. Taken together, our results illustrate the need to examine interacting cumulative effects of multiple landscape‐scale processes affecting species distributions and driving conservation actions.

## 5 CAVEATS AND DATA LIMITATIONS

Our design did not sample prey availability (e.g., Krebs et al., 2004; Lofroth & Ott, 2007), but data from Muhly, Semeniuk, Massolo, Hickman, and Musiani (2011) in the study area suggest an abundance of wolverine prey. Harvest pressure also affects wolverine survivorship (Krebs et al., 2004), but Alberta lacks a robust measure of trap effort (Webb et al., 2013). There is a harvest quota of one wolverine and one accidental wolverine per trapline per season (Alberta Fish and Wildlife 2008); our findings suggest a conservative approach to harvest may be warranted along Alberta's east slopes.

Our regionally comprehensive landscape data may not sample smaller‐scale avoidance of specific, local human activity, also suggested to influence wolverine den selection (May et al., 2012; Heinemeyer and Squires 2014). Including localized human activity may improve wolverine models. However, confidence in our results is bolstered by the uniquely large study area and sample size, high probability of detection (Fisher & Bradbury, 2014), and concordance with neighboring studies (Fisher et al., 2013). As with all landscape studies, the signals we detected are scale dependent (Holland et al., 2004; Levin, 1992). Our results reflect the extent of our study area and the gradients of the explanatory variables within it. If examining at smaller scales (e.g., within the NPC), the signal is likely to change.

## 6 CONCLUSIONS

Species distribution emerges from multiple ecological processes occurring in tandem, so it is natural that multiple forms of ecological change—such as landscape and climate change—alter this distribution. In our case, climate variables and landscape change cumulatively best‐explained wolverine distribution and spatial declines in the Canadian Rocky Mountains. This finding is likely not limited to wolverines, and emerging research supports interacting effects of climate and landscape change, with each mechanism varying in their relative weights. Notably although, a recent analysis of threats to species listed under the IUNC revealed that, regardless of the stressor or the species, overexploitation and agriculture have the greatest current impact (Maxwell et al., 2016)—greater than climate change. With paramount increases in human land‐use activities such as forest loss (e.g., Global Forest Watch Canada 2014) occurring across vast spatial scales, our findings underscore the importance of incorporating multiple mechanisms of change into spatial studies and conservation threats assessment, including the immediate direct and indirect impacts of human disturbance, to improve applied ecology research outcomes and species at risk recovery planning.

## CONFLICT OF INTEREST

None declared.

## AUTHORS’ CONTRIBUTIONS

Nicole Heim, Jason Fisher, Anthony Clevenger, and John Paczkowski conceived the ideas and designed methodology; Nicole Heim, Anthony Clevenger, and John Paczkowski collected the data; Nicole Heim and Jason Fisher analyzed the data; Nicole Heim led the writing of the manuscript. All authors contributed critically to the drafts and gave final approval for publication.
