# Supplementary material for: Cumulative effects of climate and landscape change drive spatial distribution of Rocky Mountain wolverine (Gulo gulo L.)
Source: Ecol Evol. 2017 Sep 21;7(21):8903–14. doi: 10.1002/ece3.3337 (PMC5677488; doi:10.1002/ece3.3337)

Appendix S1 – Maps survey locations with wolverine detections (black points) and non-detections (grey points) across study area, with few detections occurring outside of the National Parks Complex.

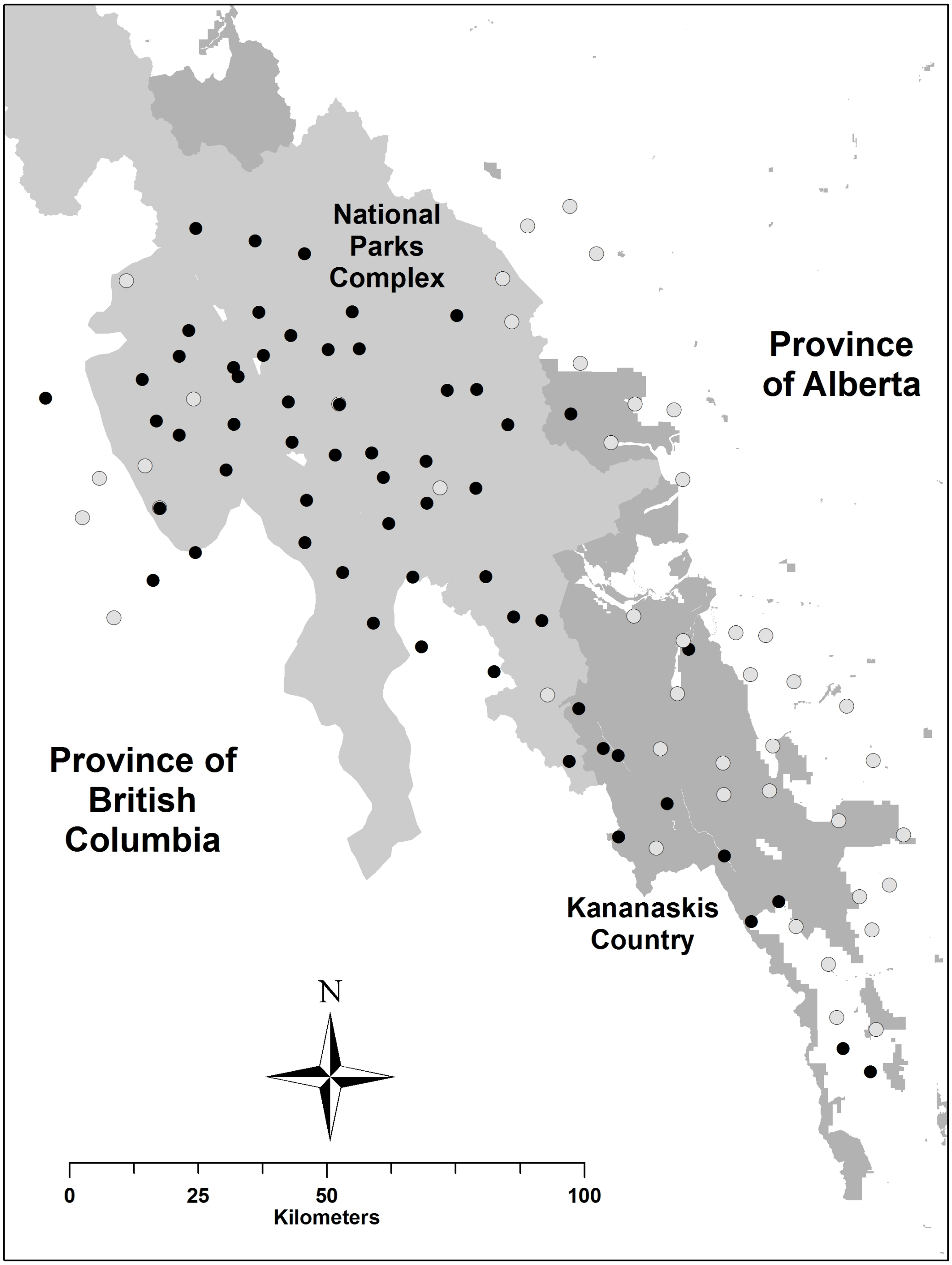

Supplement: Supplementary file 1 [file ECE3-7-8903-s001.pdf]
