# Supplementary material for: Cumulative effects of climate and landscape change drive spatial distribution of Rocky Mountain wolverine (Gulo gulo L.)
Source: Ecol Evol. 2017 Sep 21;7(21):8903–14. doi: 10.1002/ece3.3337 (PMC5677488; doi:10.1002/ece3.3337)

Appendix S2 – Shows correlation coefficient (r^2) matrix of landcover variables (quantified using a10-km radius) hypothesized to explain wolverine occurrence in the south-central region of the Canadian Rocky Mountains.

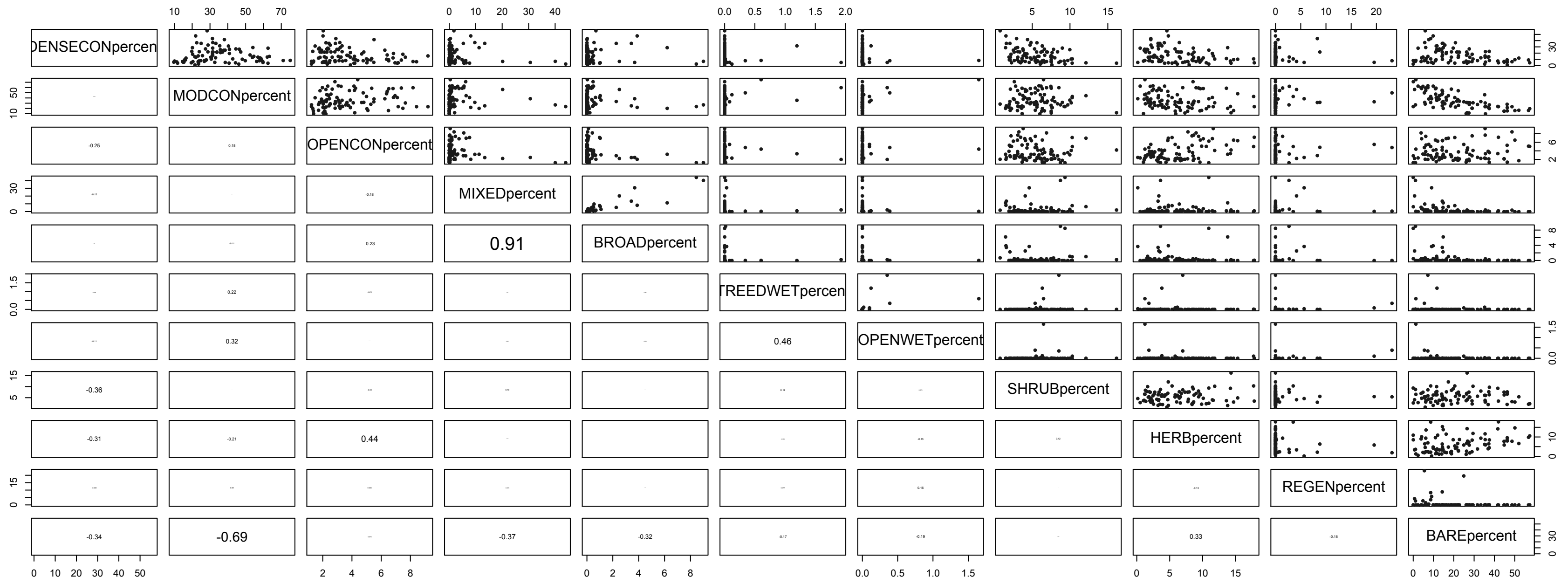

Supplement: Supplementary file 2 [file ECE3-7-8903-s002.pdf]
